# Does attending psychiatry teaching lectures change attitude of medical students towards people with mental illness? A longitudinal survey from nepal

**DOI:** 10.1192/bjo.2021.443

**Published:** 2021-06-18

**Authors:** Suresh Thapaliya, Shizu Singh, Bharat Goit, Sandesh Sawant, Anoop Krishna Gupta

**Affiliations:** 1 Kent and Medway NHS and Social Care Partnership Trust; 2 National Medical College and Teaching Hospital

## Abstract

**Aims:**

The study aims to compare the attitude of early clinical year medical students towards people with mental illness at the beginning and the end of their psychiatry teaching schedule. It hypothesizes that long exposure to psychiatry lectures can help to reduce the negative attitude.

**Background:**

Health professionals are also known to harbour negative attitude towards people with mental illness. Reducing stigma among medical students is crucial to shape the attitude of future health professionals towards people with mental illness. However, the effect of Psychiatry training on the attitude of the medical students shows mixed results.

**Method:**

It was a prospective longitudinal study conducted among fourth year medical students affiliated with a teaching hospital in Southern Nepal as an initiative to improve quality of Psychiatry training for medical students. The students who gave their consent for participation were assessed for their attitude at the beginning, after the first two introductory lectures and at the end of the Psychiatry lecture-based teaching schedule (36 two weekly lectures in 5 month period), using self-administered 16-item Mental Illness Clinician's Attitudes Scale (MICA-2) ‘medical students version’ questionnaire in English language. Permission was taken from the author of the study to use the scale. IRB approval was taken prior to the study.

**Result:**

A total of 95 (approx. 67%) students participated in the study. At the first follow-up i.e. second week, (n = 85), there was no significant difference in negative attitude as assessed by MICA score (p = 0.47). However, at six months follow-up (n = 82), the negative attitude significantly differed compared to the baseline (p < 0.001).

**Conclusion:**

While brief lectures about mental illness can provide some knowledge about mental illness, long term exposure to psychiatry lectures can reduce attitude of medical students on people with mental illness. Hence, it is also crucial to incorporate academic contents that reduce negative attitude about people with mental illness.

Financial declaration: The study was self-funded by the department of Psychiatry at National Medical College and Teaching Hospital, Parsa, Nepal.

